# An iron corrosion-assisted H_2_-supplying system: a culture method for methanogens and acetogens under low H_2_ pressures

**DOI:** 10.1038/s41598-020-76267-z

**Published:** 2020-11-05

**Authors:** Souichiro Kato, Motoko Takashino, Kensuke Igarashi, Hanako Mochimaru, Daisuke Mayumi, Hideyuki Tamaki

**Affiliations:** 1 Bioproduction Research Institute, National Institute of Advanced Industrial Science and Technology (AIST), 2-17-2-1 Tsukisamu-Higashi, Toyohira-ku, Sapporo, Hokkaido 062-8517 Japan; 2 Division of Applied Bioscience, Graduate School of Agriculture, Hokkaido University, Kita-9 Nishi-9, Kita-ku, Sapporo, Hokkaido 060-8589 Japan; 3 Institute for Geo-Resources and Environment, Geological Survey of Japan, AIST, 1-1-1 Higashi, Tsukuba, 305-8567 Japan; 4 Bioproduction Research Institute, AIST, 1-1-1 Higashi, Tsukuba, 305-8567 Japan

**Keywords:** Environmental microbiology, Archaeal physiology, Microbiology techniques

## Abstract

H_2_ is an important fermentation intermediate in anaerobic environments. Although H_2_ occurs at very low partial pressures in the environments, the culture and isolation of H_2_-utilizing microorganisms is usually carried out under very high H_2_ pressures, which might have hampered the discovery and understanding of microorganisms adapting to low H_2_ environments. Here we constructed a culture system designated the “iron corrosion-assisted H_2_-supplying (iCH) system” by connecting the gas phases of two vials (one for the iron corrosion reaction and the other for culturing microorganisms) to achieve cultures of microorganisms under low H_2_ pressures. We conducted enrichment cultures for methanogens and acetogens using rice paddy field soil as the microbial source. In the enrichment culture of methanogens under canonical high H_2_ pressures, only *Methanobacterium* spp. were enriched. By contrast, *Methanocella* spp. and *Methanoculleus* spp., methanogens adapting to low H_2_ pressures, were specifically enriched in the iCH cultures. We also observed selective enrichment of acetogen species by the iCH system (*Acetobacterium* spp. and *Sporomusa* spp.), whereas *Clostridium* spp. predominated in the high H_2_ cultures. These results demonstrate that the iCH system facilitates culture of anaerobic microorganisms under low H_2_ pressures, which will enable the selective culture of microorganisms adapting to low H_2_ environments.

## Introduction

Molecular hydrogen (H_2_) is an important intermediary metabolite and an energy carrier in anaerobic environments^1–3^. Because H_2_ is rapidly turned over in natural anaerobic environments, it occurs at very low partial pressures of only a few to several tens of pascals (Pa)^4^. In conventional studies, however, culture and isolation of H_2_-utilizing microorganisms have commonly been performed under high H_2_ partial pressures (100 kPa or more). Under such laboratory conditions, it is difficult to draw conclusions about the ecophysiology of H_2_-utilizing microorganisms in their natural environment, nor can microorganisms that have adapted to conditions with low H_2_ be isolated. In fact, the presence of uncultured H_2_-utilizing methanogens and acetogens in anaerobic environments where H_2_ concentrations are estimated to be quite low (i.e., environments with low available organic matter, including subsurface environments, peat soils, and deep-sea sediments) has been inferred by molecular environmental analyses such as metagenomics^5–7^.

Because H_2_ supplied at low partial pressure is rapidly consumed, sufficient microbial growth cannot be obtained in conventional batch culture systems. To date, several research groups have developed methods that can continuously supply H_2_ at low partial pressure to elucidate ecophysiology of hydrogenotrophic methanogens in low H_2_ environments. Morgan et al.^8^ reported a low-H_2_ culture of a hydrogenotrophic methanogen by using a continuous culture system with a continuous influx of a mixed gas containing H_2_. By using this system, the authors found that the expression of some metabolic enzymes in the methanogenic pathway is regulated by H_2_ concentration. Similar methods have frequently been utilized in subsequent studies on low H_2_ responses of hydrogenotrophic methanogens^9,10^. Sakai et al.^11^ developed the “coculture method”, in which methanogens are cocultured with heterotrophic H_2_-producing bacteria to achieve a continuous supply of H_2_ at low concentration. The coculture method enabled selective enrichment of uncultured hydrogenotrophic methanogens that were expected to adapt to low H_2_ pressures, which finally resulted in the isolation of phylogenetically novel methanogens such as *Methanocella* spp. and *Methanolinea* spp.^12,13^. The coculture method has also been employed to analyze physiological responses of methanogens to low H_2_ pressures^14,15^. Although these methods yielded laboratory cultures under low H_2_ pressures, several issues still need to be addressed. The continuous gas influx process cannot be carried out in parallel with a large number of cultures because it requires relatively complex systems, including large- or small-scale reactors and gas supply devices. Although the coculture method only requires simple systems, which makes it suitable for enrichment cultures, it cannot be directly utilized for isolation of H_2_-utilizing microorganisms because it relies on coexistence with fermentative bacteria. Furthermore, it cannot be excluded that metabolites other than H_2_ (e.g., organic acids such as acetate) affect the growth of H_2_-utilizing microorganisms.

In this study, we aimed to develop a simple method capable of selective culture of microorganisms under low H_2_ pressures. The reaction on which we focused was the corrosion of metallic iron in anoxic solution: Fe^0^ + 2H^+^  ↔ Fe^2+^  + H_2_. The concept of culturing microorganisms using H_2_ derived from iron corrosion has already been reported^16,17^. The authors demonstrated that hydrogenotrophic methanogens can be cultured using H_2_ derived from metallic iron as sole energy source. However, this method has not been applied to culture microorganisms adapted to low H_2_ environments. Considering that iron corrosion proceeds very slowly in anoxic and circumneutral solution because of the small difference in the standard redox potentials of Fe^0^ oxidation (E_0_ʹ ≈ − 0.47 V) and the reduction of protons to generate H_2_ (E_0_ʹ ≈ − 0.41 V), we can expect that H_2_ supply via iron corrosion is suitable to culture hydrogenotrophic microorganisms under low H_2_ pressures. Here we report that a culture system based on iron corrosion reactions has been successfully used for the selective enrichment of hydrogenotrophic methanogens and acetogens, which have the potential to adapt to environments with very low H_2_ content.

## Results and discussion

### Validation of continuous H_2_ supply by iron corrosion

**Figure 1:**
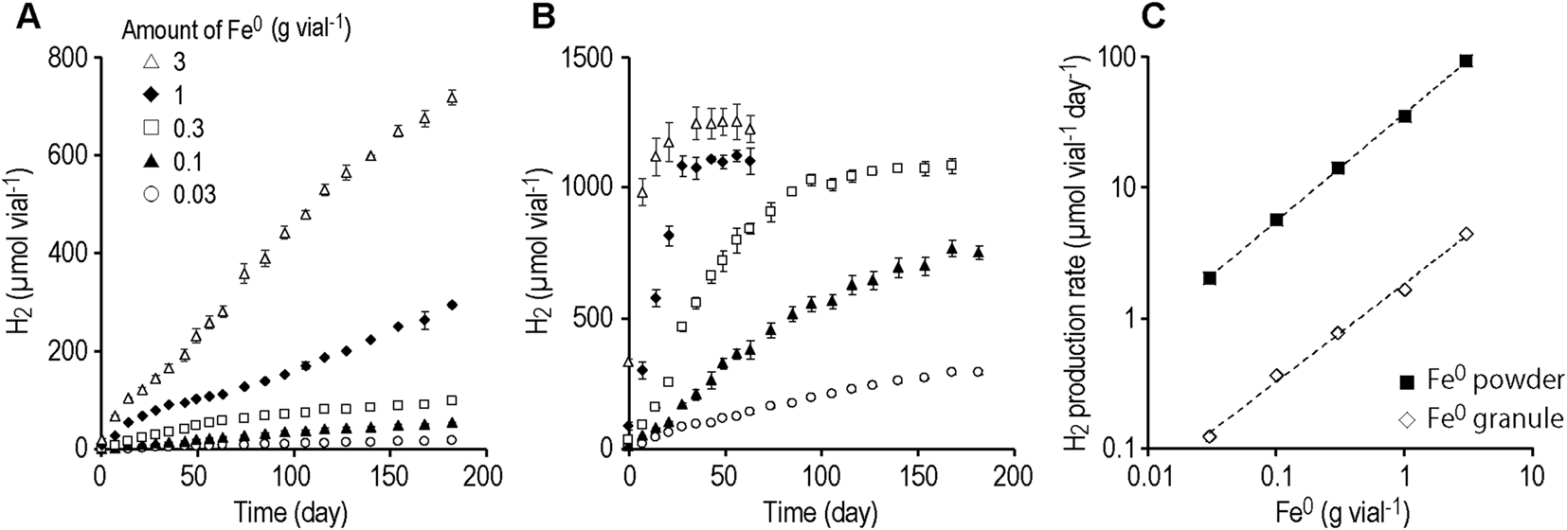
Continuous production of H_2_ by the corrosion reaction of Fe^0^ granules (1–2 mm) (**A**) and Fe^0^ powder (< 45 µm) (**B**). (**C**) H_2_ production rates calculated from the data in A and B.

Because the growth of microorganisms is considered to be very slow under low H_2_ pressures, the culture system requires a continuous supply of H_2_ over a long period of time. Hence, we first determined whether H_2_ can be continuously supplied for a long time by the iron corrosion reaction. Furthermore, to develop a culture system capable of regulating the H_2_ supply rate, we added various amounts of Fe^0^ with different particle sizes (Fe^0^ granules with a diameter of 1–2 mm or Fe^0^ powder with a diameter < 45 µm) to the anoxic buffer solution and determined the H_2_ production rates (Fig. 1).

We observed continuous H_2_ production for more than 6 months in the vials supplemented with Fe^0^ granules (Fig. 1A). The H_2_ production rates were almost proportional to the amount of Fe^0^ granules added (0.03 to 3 g vial^−1^) and were in the range of 0.12 to 4.4 μmol vial^−1^ day^−1^ (Fig. 1C). With Fe^0^ powder, H_2_ production ceased after approximately 2 weeks, 1 month, and 3 months in the vials supplemented with Fe^0^ at 3, 1, and 0.3 g vial^−1^, respectively (Fig. 1B). The highest H_2_ accumulation was approximately 1000 μmol vial^−1^, corresponding to approximately 50 kPa. We assume that H_2_ production ceased for thermodynamic reasons (increase in H_2_ partial pressure and decrease in proton concentration).The H_2_ production rates were also proportional to the amount of Fe^0^ powder added, with a range of 2.0 to 93 μmol vial^−1^ day^−1^ (Fig. 1C). Since the specific surface area of the spherical material is inversely proportional to the diameter, the Fe^0^ powder (< 45 µm) has a surface 20 times larger than the Fe^0^ granule (1–2 mm), if used particles are assumed to be spherical. The H_2_ production from Fe^0^ powder was 16–22 times greater than from Fe^0^ granule (Fig. 1C), suggesting that surface area is the major determinant of H_2_ production rate. These results demonstrate that it is possible to supply H_2_ over a long period of time by utilizing the iron corrosion reaction, and also that it is possible to regulate the rate of H_2_ supply by altering the size and amount (i.e., total surface area) of Fe^0^ particles.

### Development of a culture system with low H_2_ pressures

**Figure 2:**
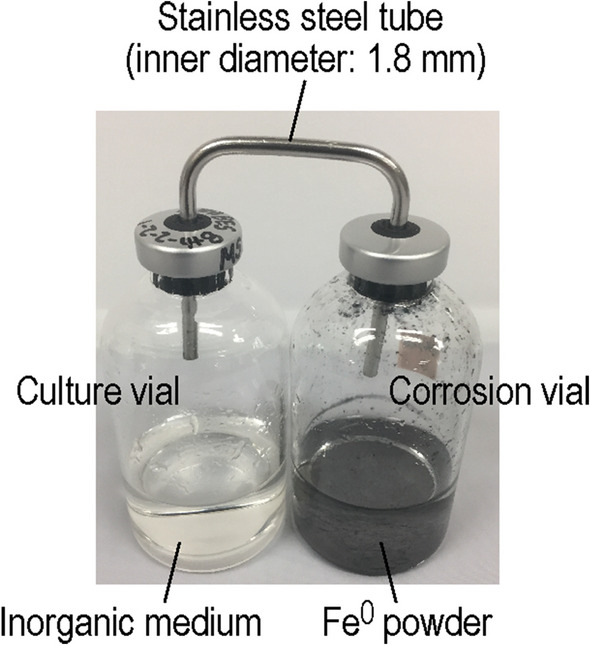
The iron corrosion-assisted H_2_-supplying (iCH) system. The vial on the left is for culture of microorganisms and that on the right is for the iron corrosion (i.e., H_2_ production) reaction. The gas phases of the two vials are connected by a stainless steel tube to allow diffusion of H_2_ produced in the corrosion vial.

It has been reported that H_2_-utilizing anaerobic microorganisms grow on H_2_ derived from iron corrosion^16–20^. If microorganisms are cultured in the coexistence with Fe^0^, however, some undesirable phenomena can occur. For example, the iron corrosion reaction produces a high concentration of ferrous iron and induces an increase in pH due to the consumption of protons. Furthermore, it can promote growth of anaerobic microorganisms that use Fe^0^ itself as the energy source^18,21,22^. These phenomena likely hamper the culture of target microorganisms. We therefore constructed a culture system in which the gas phases in two vials (one for the culture of microorganisms and the other for the iron corrosion reaction) are connected by a stainless-steel tube (Fig. 2), in reference to the system developed by Daniels et al.^16^. This system, hereafter referred to as the “iron corrosion-assisted H_2_-supplying (iCH) system”, was expected to allow the culture of microorganisms under low H_2_ pressures while avoiding the unfavorable effects of the iron corrosion reaction.

### Pure culture of a hydrogenotrophic methanogen in the iCH system

**Figure 3:**
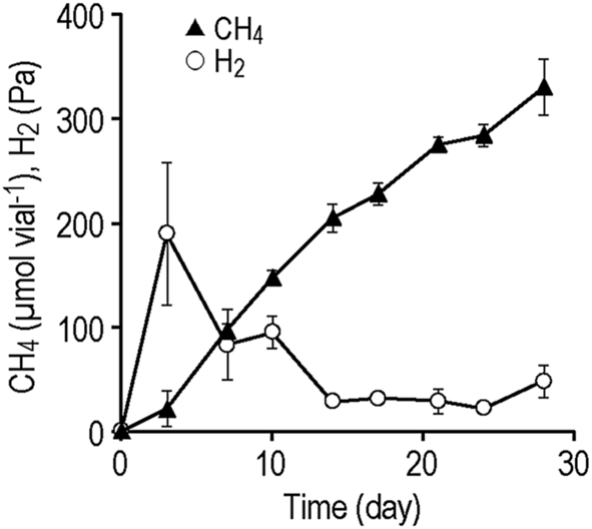
The amount of CH_4_ and the partial pressure of H_2_ during the culture of *Methanobacterium formicicum* with the iron corrosion-assisted H_2_-supplying (iCH) system supplemented with 1 g Fe^0^ powder. Data are presented as the means of three independent cultures, and error bars represent standard deviations.

We evaluated the capability of the iCH system to culture microorganisms under low H_2_ pressures using a hydrogenotrophic methanogen *Methanobacterium formicicum*, which is known to be able to grow under both high and low H_2_ partial pressures^23^, as a model strain. We inoculated the iCH system with *M. formicicum* using Fe^0^ powder at 1 g vial^–1^ as the H_2_ source, and monitored the amounts of CH_4_ and H_2_ in the gas phase (Fig. 3). We observed continuous CH_4_ production by *M. formicicum* in the iCH system for almost 1 month. Although there was accumulation of H_2_ at the beginning of the culture (around 190 Pa at day 3, corresponding to 7.4 µmol vial^–1^), the partial pressure of H_2_ subsequently decreased and remained extremely low (30 to 50 Pa after day 14). The observed H_2_ partial pressures are comparable to those observed in natural anaerobic environments and laboratory cocultures of methanogens with fermentative bacteria^4,24,25^. These results demonstrated that the iCH system is capable of long-term culture of H_2_-utilizing microorganisms under low H_2_ pressures.

### Enrichment cultures of hydrogenotrophic methanogens using the iCH system

We performed enrichment cultures of methanogens to demonstrate the capability of the iCH system to selectively culture microorganisms adapted to low H_2_ pressures. We used rice paddy field soil as the microbial source, because it is known as a low H_2_ environment^4^ and was the isolation source of *Methanocella* spp. that is known to adapt to low H_2_ environments^12,26,27^. In addition to the iCH system (with Fe^0^ powder at 1 g vial^–1^ as the H_2_ source), we set up conventional cultures under high H_2_ pressures (160 kPa of H_2_ in the gas phase, hereafter referred to as “high H_2_ enrichments”) and cultures under iron corrosion conditions (microorganisms cultured in the same vial with 1 g vial^–1^ of Fe^0^ powder, hereafter referred to as “Fe^0^ enrichments”) as control experiments.

**Figure 4:**
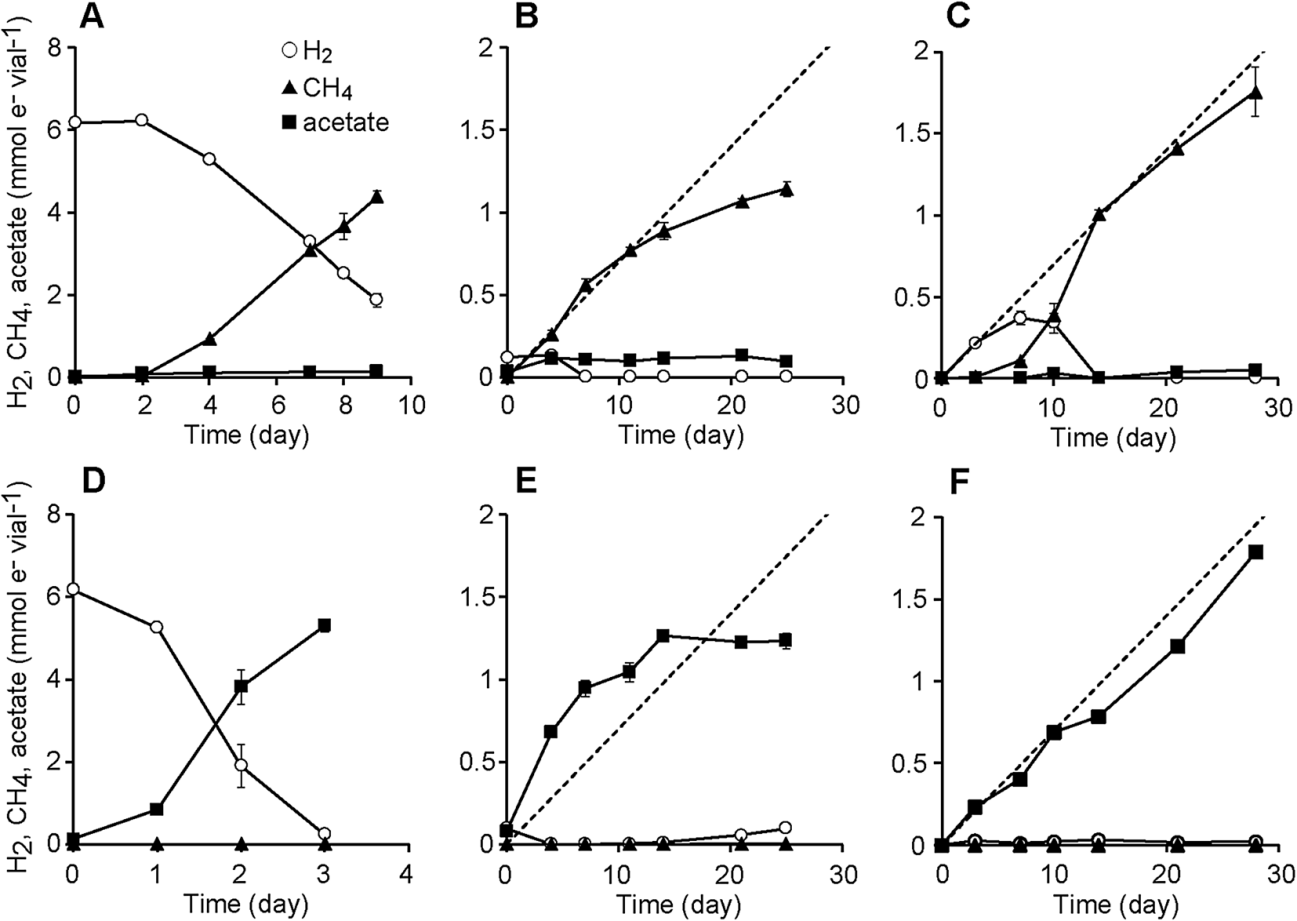
Production and consumption of H_2_, CH_4_, and acetate in enrichment cultures. (**A**–**C**) Enrichment cultures for methanogens (supplemented with rifampicin). (**D**–**F**) Enrichment cultures for acetogens (supplemented with 2-bromoethanesulphonate [BES]). (**A**,**D**) “High H_2_ enrichments” supplemented with 160 kPa of H_2_ in the gas phase. (**B**,**E**) “Fe^0^ enrichments” in which microorganisms were cultured in the same vial with 1 g vial^−1^ of Fe^0^ powder. (**C**,**F**) Enrichment cultures with the iron corrosion-assisted H_2_-supplying (iCH) system, in which H_2_ was continuously supplied by the iron corrosion reaction. For ease of comparison, the amounts of all metabolites are represented as electron equivalents in units of mmol e^–^ per culture vial, using the respective half reaction formulas; 2H^+^ + 2e^−^  ↔ H_2_, HCO_3_^−^ + 9H^+^ + 8e^−^ ↔ CH_4_ + 3H_2_O, and 2HCO_3_^−^ + 9H^+^ + 8e^−^ ↔ CH_3_COO^−^ + 4H_2_O. The volumes of the liquid and gaseous headspace were 20 and 48 ml, respectively. The broken lines represent the rate of H_2_ production via the iron corrosion reaction calculated from the data shown in Fig. 1C (35 µmol vial^−1^ day^−1^).

Hydrogenotrophic methanogens were enriched in inorganic medium supplemented with rifampicin to suppress growth of bacteria. After three successive subcultures, the metabolic products (CH_4_ and H_2_) were analyzed during incubation (Fig. 4A–C). In the high H_2_ enrichments, we observed CH_4_ production almost equal to the theoretical value calculated from the consumption of H_2_ (Fig. 4A). In the Fe^0^ enrichments, we observed CH_4_ production comparable to the theoretical value (broken line in Fig. 4B) calculated from the H_2_ production via iron corrosion in the early phase of the incubation (day 0–14). However, CH_4_ production levelled off in the later phase (after day 14). The pH of the culture solution of Fe^0^ enrichments increased from 7.0 to around 8.1 during the incubation, whereas the pH of the culture solution remained around 7.0 in the other enrichments. The increase in pH, and possibly the increase in concentration of ferrous iron, might have inhibited the growth of methanogens in the Fe^0^ enrichments. In the iCH enrichments, CH_4_ was produced almost proportional to the theoretical value (broken line in Fig. 4C) without levelling off. We observed accumulation of H_2_ at the beginning of culture (around day 10), which then decreased below the detection limit (< 10 Pa). These results indicate that enrichment cultures of methanogens under low H_2_ pressures were achieved in the iCH system.

### Microbial community analysis of the methanogenic enrichment cultures

To confirm the capability of the iCH system to specifically enrich microorganisms adapting to low H_2_ pressures, we assessed the microbial community structures of the enrichment cultures and the inoculum soil by high throughput sequencing analysis of 16S rRNA gene amplicons. A total of 56,388 16S rRNA gene reads (3744–5531 reads per sample) were retrieved and classified into 2949 operational taxonomic units (OTUs) using a 97% sequence identity cut-off. The microbial composition is displayed in Fig. S1A. All OTUs dominant in the enrichment cultures (relative abundance > 3% in at least one enrichment culture) had low abundance in the inoculum soil (~ 1.2%), suggesting that H_2_-utilizing microorganisms were sufficiently enriched. Principal component analysis was performed to quantitatively evaluate the similarity of microbial community structures of each sample (Fig. S1B). The results showed that the microbial community patterns of the duplicate enrichment cultures were very similar, and that the microbial community composition differs between the different experimental setups. Therefore, for further analysis we used the average values of community analysis data of the duplicate cultures.

**Figure 5:**
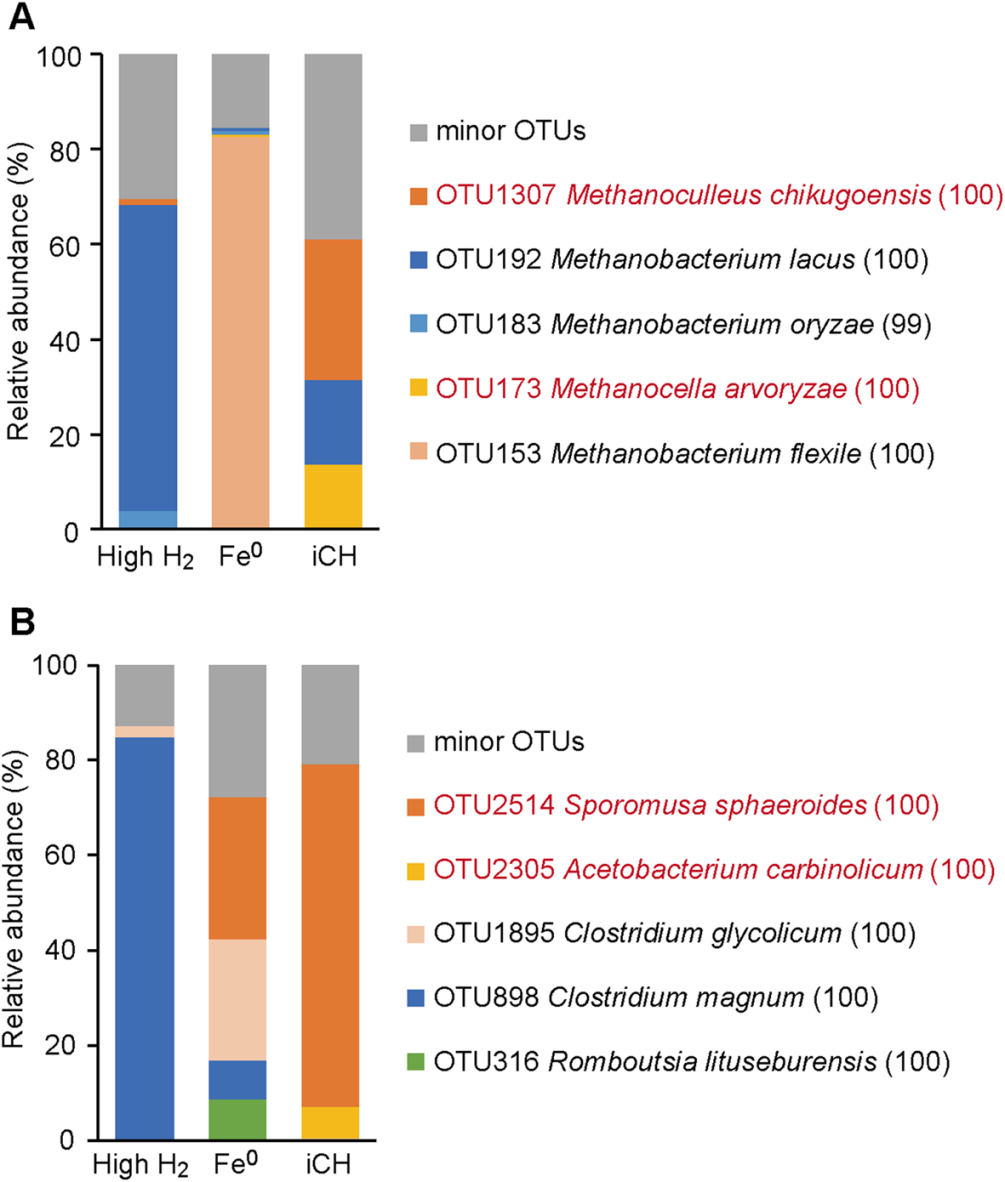
Relative abundance of the OTUs recovered from the enrichment cultures for methanogens (**A**) and acetogens (**B**). The dominant OTUs (> 3% in at least one enrichment) and their closest relatives (sequence identity, %) are shown in the legends. The OTUs specifically enriched in the iron corrosion-assisted H_2_-supplying (iCH) system are highlighted in red. Minor OTUs, ≤ 3% in all enrichments.

We plotted the relative abundances of the dominant archaea (> 3% under at least one set of culture conditions) in the methanogenic enrichments (Fig. 5A). Different types of methanogens were enriched depending on the culture conditions. In the high H_2_ enrichments, two OTUs closely related to *Methanobacterium* spp. (OTU183, with 99% identity to *Methanobacterium oryzae* and OTU192, with 100% identity to *Methanobacterium lacus*) predominated. By contrast, OTU173 and OTU1307 (100% identity to *Methanocella arvoryzae* and *Methanoculleus chikugoensis*, respectively) were specifically enriched in the iCH cultures. The genera *Methanocella* and *Methanoculleus* have been frequently detected as dominant hydrogenotrophic methanogens in various anaerobic environments with low H_2_ concentrations, including rice paddy fields, peat bogs, marine and freshwater sediments, and subsurface environments^28–33^. Furthermore, methanogens closely related to these genera have been selectively enriched from rice paddy field soils and marine/freshwater sediments under the low H_2_ pressures achieved by the coculture method^11^. These findings suggest that the iCH system can selectively culture hydrogenotrophic methanogens adapting to low H_2_ pressures. By contrast, a different type of methanogen (OTU153, with 100% identity to *Methanobacterium flexile*) was enriched in the Fe^0^ enrichment cultures. This suggests that factors other than H_2_ concentration (e.g., increase in pH and/or high concentration of ferrous iron) were the main selective pressures in the Fe^0^ enrichments.

### Acetogen enrichment culture and its microbial community analysis

In addition to methanogenic archaea, acetogenic bacteria are also one of the important H_2_-utilizing microorganisms in anaerobic environments^34,35^. Hence, we set up enrichment cultures of H_2_-utilizing acetogens with inorganic medium supplemented with 2-bromoethanesulphonate (BES) to inhibit methanogens. We followed the transitions of metabolites during the incubations of enrichment cultures of acetogens in the high H_2_, Fe^0^, and iCH cultures (Fig. 4D–F). As described below, the trends were similar to those observed with the enrichment of methanogens. In the high H_2_ enrichments, we observed acetate production with concomitant consumption of H_2_, and the H_2_ consumption rate was much higher than in enrichment cultures of methanogens (Fig. 4D). In the Fe^0^ enrichments, we observed acetate production comparable to the theoretical value from day 0 to day 14, after which acetate production ceased (Fig. 4E), suggesting that the Fe^0^ cultures have inhibitory effects on acetogens as observed in the methanogenic enrichments. In the iCH enrichments, acetate was produced at a rate comparable with theoretical values (Fig. 4F). Although H_2_ initially accumulated in the iCH enrichments, it was below the detection limit (< 10 Pa) after day 14. These results indicate that acetogens adapting to low H_2_ pressures could be enriched in the iCH system.

We plotted the relative abundances of the dominant bacteria (> 3% under at least one set of conditions) in the enrichments for acetogens (Fig. 5B). As with the methanogen enrichments, the acetogen community structures were completely different for each culture condition. Only one phylotype (OTU898) closely related to *Clostridium magnum*, which is well known as an acetogen^33^, was enriched in the high H_2_ enrichments. In contrast, two phylotypes closely related to other acetogen species (OTU2305 and OTU2514, with 100% identities to *Acetobacterium carbinolicum* and *Sporomusa sphaeroides*, respectively) were selectively enriched in the iCH cultures. The phylotypes closely related to *Clostridium glycolicum* and *Romboutsia lituseburensis* (OTU316 and OTU1895, respectively) predominated in the Fe^0^ enrichments, in addition to OTU898 (*C. magnum*) and OTU2514 (*S. sphaeroides*), which were also detected in other enrichments. *Clostridium glycolicum* is a well-known acetogen species^34^. *Romboutsia* spp. have often been detected in enrichment cultures of H_2_-utilizing acetogens^36^, although there is no report of their acetogenic metabolism.

It has been reported that affinities for H_2_ and kinetics of H_2_ consumption differ depending on the species of acetogens, mainly due to the differences in their energy acquisition efficiencies^37^. In contrast to methanogens, however, there are only few studies of the response of acetogens to low H_2_ pressures. Generally, acetogens have lower affinity for H_2_ than methanogens for thermodynamic reasons^38^, which may be one of the reasons that the ecophysiology of acetogens under low H_2_ pressures has not attracted much attention. Our result shows that different types of acetogens can be selectively enriched under conditions with different H_2_ availability. This suggests that environmental H_2_ concentration provides an ecological niche not only for methanogens but also for acetogens.

## Conclusion

We constructed a simple system for culturing anaerobic microorganisms under low H_2_ pressures by using the iron corrosion reaction as the source of H_2_. The system, which we call the iCH system, can continuously supply H_2_ for several months, and it is possible to control the H_2_ supply rate by changing the amount and size of Fe^0^ particles. We demonstrated that the iCH system can selectively enrich anaerobic microorganisms adapting to low H_2_ pressures. Although this study focused only on methanogens and acetogens, the iCH system is applicable to cultures of other H_2_-utilizing microorganisms such as nitrate, iron, and sulfate reducers. The iCH system is also applicable to colony isolation using agar-solidified media (e.g., a roll-tube method), which is an on-going study in our research group. This culture method should enable selective enrichment and isolation of unidentified microorganisms adapting to or even specialized for low H_2_ pressures from anaerobic environments with low H_2_ availability, such as subsurface environments, peat soils, and deep-sea sediments, which would shed light on the novel ecophysiology of hydrogenotrophic microorganisms in anaerobic environments.

## Materials and methods

### Bacterial strains and culture conditions

To culture microorganisms we used a freshwater basal medium containing (per liter) 0.3 g KH_2_PO_4_, 1 g NH_4_Cl, 0.1 g MgCl_2_·6H_2_O, 0.08 g CaCl_2_·2H_2_O, 0.6 g NaCl, 2 g KHCO_3_, 0.02 g MgSO_4_·7H_2_O, 9.52 g 4-(2-hydroxyethyl)-1-piperazineethanesulfonate (HEPES), 0.1 g yeast extract, and 10 ml each of trace metal and vitamin solutions^39^. The pH of the medium was adjusted to 7.0 by adding 6 N KOH solution. *Methanobacterium formicicum* (DSM1535^T^) was cultured in the freshwater basal medium supplemented with 0.1 g l^–1^ of sodium acetate and reducing agents (0.3 g l^–1^ each of cysteine·HCl·H_2_O and Na_2_S·9H_2_O) at 37 °C without shaking under an atmosphere of 200 kPa of H_2_:CO_2_ (80:20). CH_4_ and H_2_ in the gas phases were measured using a gas chromatograph (GC-2014; Shimadzu, Kyoto, Japan) equipped with a thermal conductivity detector (for quantification of H_2_) and a flame ionization detector (for quantification of CH_4_) as described previously^40^. The concentration of acetate was determined using high-performance liquid chromatography (D-2000 LaChrom Elite HPLC system; Hitachi, Tokyo, Japan) equipped with an ion exclusion column (Aminex HPX-87H; Bio-Rad Laboratories, Hercules, CA, USA) and UV detector (L2400; Hitachi). The culture experiments were conducted in triplicate.

### The iron corrosion-assisted H_2_-supplying (iCH) system

The iCH system consists of two vials (68 ml in capacity). One vial (“corrosion vial” in Fig. 2) was filled with 20 ml of the freshwater basal medium and supplemented with Fe^0^ granules (1–2 mm, 99.98% purity; Alfa Aesar, Ward Hill, MA, USA) or Fe^0^ powder (< 45 µm, 99.9% purity; Wako Pure Chemical, Osaka, Japan). The second vial (“culture vial” in Fig. 2) was also filled with 20 ml of the freshwater basal medium. After removing the air from the medium by bubbling with N_2_:CO_2_ (80:20) gas for 5 min, the vials were sealed with butyl rubber stoppers and aluminum seals, and sterilized by autoclaving. After the cultivation vials were supplemented with reducing agents, inhibitor chemicals, and/or microorganisms, the gas phases of the two vials were connected by sterile, stainless-steel tube with an inner diameter of 1.8 mm (Swagelok, Solon, OH, USA), which was separately sterilized by autoclaving, through a guiding hole made by a gauge 18 syringe needle. Before incubation, the vials were again purged with N_2_:CO_2_ (80:20) gas for 5 min to remove trace oxygen. To confirm that the gases were uniformly diffused, CH_4_ and H_2_ in the gas phases of the two vials were measured during incubation by gas chromatography as described above.

### Enrichment cultures from rice paddy field soil

High-H_2_ and Fe^0^ enrichment cultures were performed in vials (not connected to the corrosion vial) filled with 20 ml of the freshwater basal medium supplemented with 200 kPa of H_2_:CO_2_ (80:20) gas and 1 g of Fe^0^ powder as the sole energy source, respectively. Rifampicin (final concentration, 10 µg l^–1^) and BES (final concentration, 10 mM) were supplemented to the enrichment cultures for methanogens and acetogens, respectively, from filter-sterilized stock solutions. Approximately 50 mg (wet weight) of rice paddy field soil was suspended in 200 µl of the freshwater basal medium, inoculated into the culture vial using a syringe and incubated at 30 °C without shaking. After sufficient microbial growth, 0.4 ml of culture solution was transferred to fresh media in a culture vial connected to a fresh corrosion vial. After three transfers, the enrichment cultures were subjected to chemical and phylogenetic analyses. The enrichment culture experiments were conducted in duplicate.

### Microbial community analysis

Microbial DNA was extracted with the FAST DNA Spin Kit for Soil (MP Biomedicals, Santa Ana, CA, USA) according to the manufacturer’s instructions. Partial 16S rRNA gene fragments were amplified by PCR using Phusion Hot Start II High-Fidelity DNA Polymerase (Thermo Fisher Scientific, Waltham, MA, USA) with the primer pair 515ʹF/805R^41^ prolonged by adaptor and index sequence tags^42^. PCR products were purified using a QIAquick PCR Purification Kit (Qiagen, Hilden, Germany), and were qualified and quantified using a spectrophotometer (DS-11; Denovix, Wilmington, DE, USA) and Qubit dsDNA HS Assay Kit (Thermo Fisher Scientific), respectively. The 16S rRNA gene amplicon libraries were analyzed by Illumina paired-end (2 × 301 bp) sequencing on MiSeq platform (Illumina, San Diego, CA, USA) by Hokkaido System Science Co. Ltd. (Sapporo, Japan). Raw 16S rRNA sequence data were adaptor trimmed at the 3ʹ end to remove adaptor sequences (cutadapt 1.1)^43^, quality trimmed (Trimmomatic v. 0.32; TRAILING:20 MINLEN:50), and the individual read pairs were overlapped to form single synthetic reads (fastq-join v. 1.1.2–537; 8 percent maximum difference, 6 minimum overlap; https://github.com/brwnj/fastq-join). The obtained reads were clustered with the UCLUST algorithm using a ≥ 97% sequence identity cut-off^44^ with MacQIIME 1.9.1^45^. Representative sequences of each OTU were aligned using PyNAST^46^ and chimeric sequences were removed using ChimeraSlayer^47^. Taxonomic assignment of each OTU was carried out with a dataset obtained from the Greengenes website (gg_13_8_otus; https://greengenes.secondgenome.com/)^48^. Principal component analysis was performed using MacQIIME 1.9.1^45^. The sequence data obtained in this study have been deposited in DDBJ/EMBL/GenBank under the accession numbers DRA007911 and DRA007912.

## Supplementary information

Supplementary Figure 1.
