# Supplementary material for: An iron corrosion-assisted H2-supplying system: a culture method for methanogens and acetogens under low H2 pressures
Source: Sci Rep. 2020 Nov 5;10:19124. doi: 10.1038/s41598-020-76267-z (PMC7645788; doi:10.1038/s41598-020-76267-z)
Supplement: Supplementary file 1 — Supplementary Figure 1. [file 41598_2020_76267_MOESM1_ESM.pdf]

An iron corrosion-assisted H_2-supplying system: A culture method for methanogens and acetogens under low H_2 pressures

Souichiro Kato,[1,2#] Motoko Takashino,[1] Kensuke Igarashi,[1] Hanako Mochimaru,[3] Daisuke Mayumi,[3] Hideyuki Tamaki[4]

[1] *Bioproduction Research Institute, National Institute of Advanced Industrial Science and Technology (AIST), 2-17-2-1 Tsukisamu-Higashi, Toyohira-ku, Sapporo, Hokkaido 062-8517, Japan*

[2] *Division of Applied Bioscience, Graduate School of Agriculture, Hokkaido University, Kita-9 Nishi-9, Kita-ku, Sapporo, Hokkaido 060-8589, Japan*

[3] *Institute for Geo-Resources and Environment, Geological Survey of Japan, AIST, 1-1-1 Higashi, Tsukuba 305-8567, Japan*

[4] *Bioproduction Research Institute, AIST, 1-1-1 Higashi, Tsukuba 305-8567, Japan*

[#]Address correspondence to Souichiro Kato, s.katou@aist.go.jp

Supplemental Materials

FIG S1 (A) Relative abundance of taxa identified in the enrichment cultures and the inoculum soil. The dominant OTUs (>3% in at least one sample) and their closest relatives (sequence identity, %) are shown in the legend. (B) The result of principal component analysis using the community analysis data shown in panel A. Duplicate enrichment cultures are indicated by the dotted ovals. Minor OTUs, ≤3% in all samples. Rif, rifampicin; BES, 2-bromoethanesulphonate.

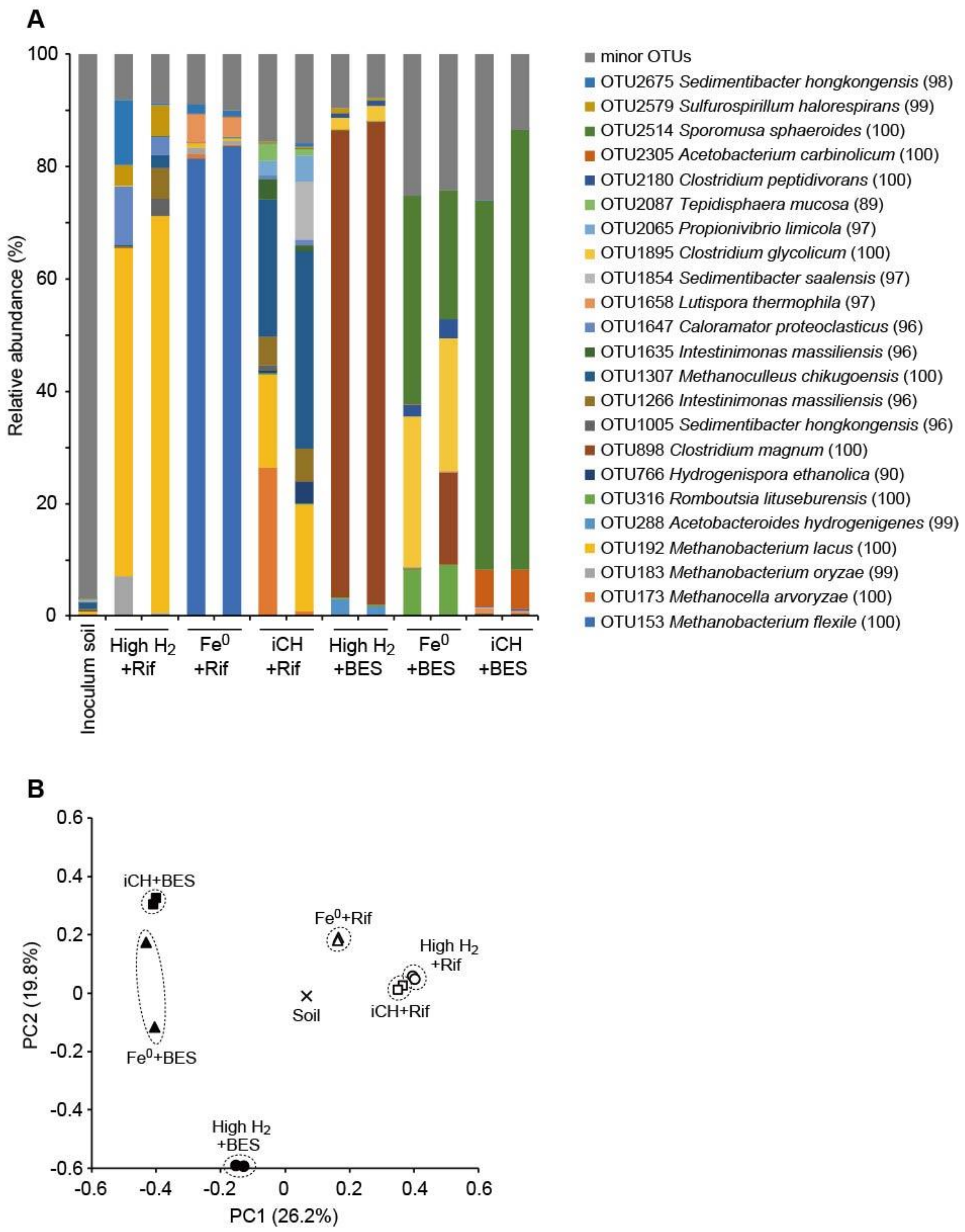
A
Relative abundance (%)
100
80
60
40
20
0
Inoculum soil
High H2 +Rif
Fe0 +Rif
iCH +Rif
High H2 +BES
Fe0 +BES
iCH +BES
minor OTUs
OTU2675 Sedimentibacter hongkongensis (98)
OTU2579 Sulfurospirillum halorespirans (99)
OTU2514 Sporomusa sphaeroides (100)
OTU2305 Acetobacterium carbinolicum (100)
OTU2180 Clostridium peptidivorans (100)
OTU2087 Tepidisphaera mucosa (89)
OTU2065 Propionivibrio limicola (97)
OTU1895 Clostridium glycolicum (100)
OTU1854 Sedimentibacter saalensis (97)
OTU1658 Lutispora thermophila (97)
OTU1647 Caloramator proteoclasticus (96)
OTU1635 Intestinimonas massiliensis (96)
OTU1307 Methanoculleus chikugoensis (100)
OTU1266 Intestinimonas massiliensis (96)
OTU1005 Sedimentibacter hongkongensis (96)
OTU898 Clostridium magnum (100)
OTU766 Hydrogenispora ethanolica (90)
OTU316 Romboutsia lituseburensis (100)
OTU288 Acetobacteroides hydrogenigenes (99)
OTU192 Methanobacterium lacus (100)
OTU183 Methanobacterium oryzae (99)
OTU173 Methanocella arvoryzae (100)
OTU153 Methanobacterium flexile (100)
B
PC2 (19.8%)
PC1 (26.2%)
0.6
0.4
0.2
0
-0.2
-0.4
-0.6
-0.6
-0.4
-0.2
0
0.2
0.4
0.6
iCH+BES
Fe0+Rif
High H2 +Rif
Soil
iCH+Rif
Fe0+BES
High H2 +BES
